# Supplementary material for: A Systematic Literature Review of the Impact of Climate Change on the Global Demand for Psychiatric Services
Source: Int J Environ Res Public Health. 2023 Jan 9;20(2):1190. doi: 10.3390/ijerph20021190 (PMC9858749; doi:10.3390/ijerph20021190)
Supplement: Supplementary file 1 [file ijerph-20-01190-s001.zip › ijerph-2068108-supplementary.docx]

Supplementary Materials


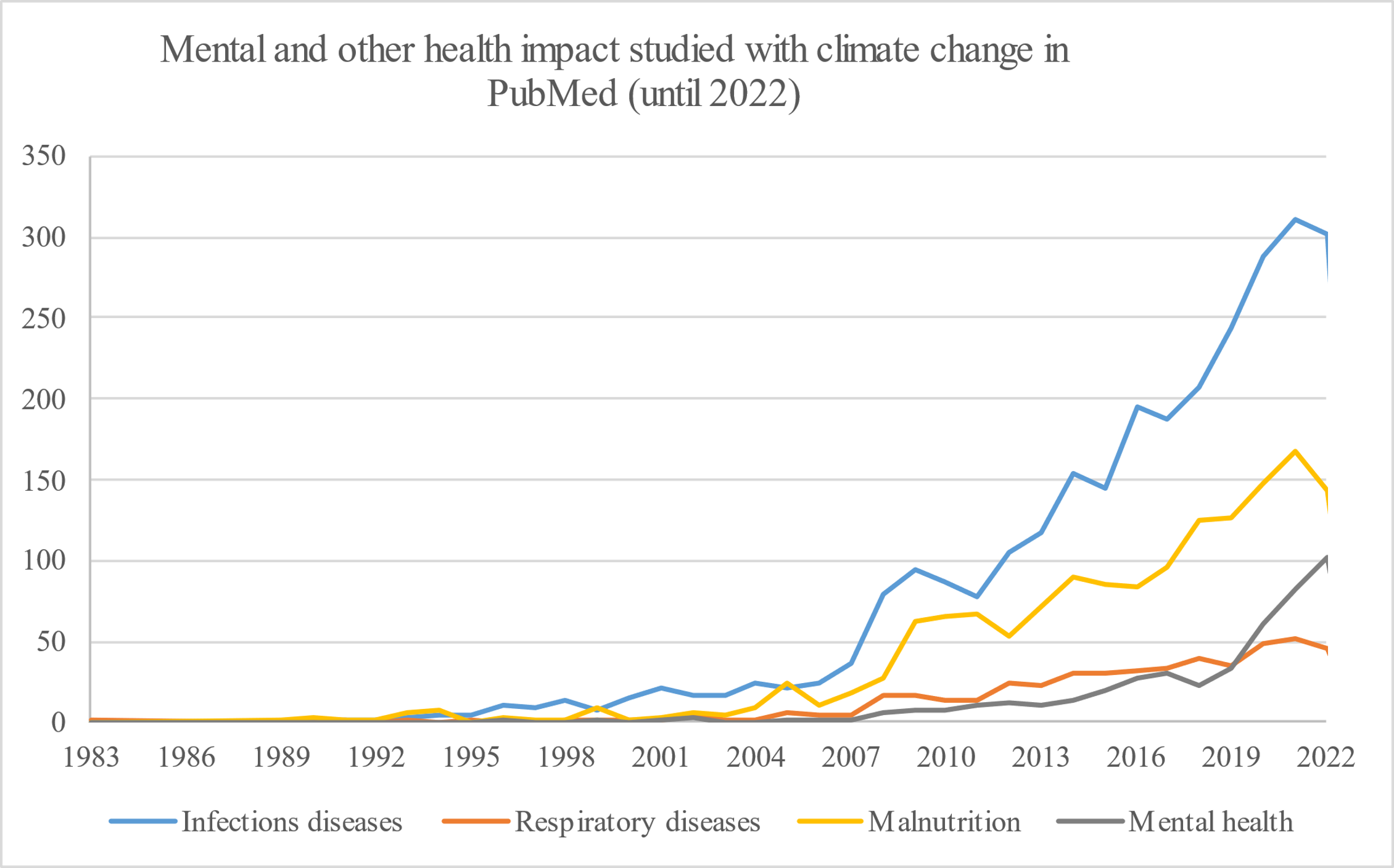


**Figure S1.** Mental and other health impacts studied with climate change in PubMed (until 2022). Source: authors, based on a previous study from Verner et al. (2016). Detailed keywords used for the PubMed research can be found in the Supplementary Material Table S2.

**Table S2**. Search terms used on PubMed for the elaboration of Figure S1.

| **The search terms used for malnutrition, respiratory and infectious diseases were retrieved identically from Verner et. al. (2016): We adapted them for the mental health research, but ensured to follow the same pattern. The different keywords are, as follow:** | |
| --- | --- |
| **Infectious diseases (from Verner et. al., 2016)** | ("climate change" OR "global warming" OR "climate variability" OR "greenhouse effect") **AND** ("Malaria" OR "Dengue" OR "Encephalitis" OR "Tick-Borne" OR "Tick-Borne Diseases" OR "Chikungunya Fever" OR "West Nile Fever" OR "Rift Valley fever virus" OR "Encephalitis, Japanese" OR "Haemorrhagic Fever with Renal Syndrome" OR "Hantaan virus" OR "Plague" OR "Lyme Disease" OR "Vibrio" OR "Cholera" OR "Salmonella" OR "Campylobacter" OR "Diarrhea" OR "Salmonella Infections" OR "Campylobacter Infections" OR "Enterovirus Infections" OR "Enterovirus" OR "Coxsackievirus Infections" OR "Hand, Foot and Mouth Disease" OR "Rotavirus" OR "Rotavirus Infections" OR "Harmful Algal Bloom" OR "Dysentery" OR "Schistosomiasis") |
| Malnutrition (from Verner et. al., 2016): | ("climate change" OR "global warming" OR "climate variability" OR "greenhouse effect") **AND** ("Nutritional Status" OR "Fetal Nutrition Disorders" OR "Child Nutrition Disorders" OR "Infant Nutrition Disorders" OR "Malnutrition" OR "Wasting Syndrome" OR "Thinness" OR "Food Supply") |
| Respiratory diseases (from Verner et. al., 2016) | ("climate change" OR "global warming" OR "climate variability" OR "greenhouse effect") **AND** ("Respiratory Tract Diseases" OR "Respiration Disorders" OR "Hypersensitivity" OR "Allergy and Immunology" OR "Rhinitis, Allergic" OR "Rhinitis, Allergic, Seasonal" OR "Conjunctivitis, Allergic" OR "Dermatitis" OR "Eczema") |
| Mental health (adapted from Verner et. al., 2016) | ("climate change" OR "global warming" OR "climate variability" OR "greenhouse effect") **AND** ("psychiatric disorder" OR "mental disorder" OR "suicide" OR "suicide attempt" OR "depression" OR "mania" OR "schizophrenia" OR "dementia" OR "substance abuse” OR “bipolar disorder” OR “psychosis” OR “organic disorder” OR “anxiety disorder” OR “neurotic disorder” OR “neurocognitive disorder” OR “Post-traumatic stress disorder”) |

**Table S3**. Detailed search terms and its sources used for **this review**

| **Detailed search terms and its sources used for this review** |
| --- |
| (“climate change"[Mesh] **OR** "global warming"[Mesh] **OR** "heatwave"[relevant CC variable on MH] **OR** "hot temperature"[Mesh] **OR** “natural disaster”[Mesh]) **AND** ("psychiatric disorder"[ICD-10] **OR** "mental disorder"[Mesh] **OR** "suicide"[Mesh and ICD-10] **OR** "suicide attempt"[Mesh and ICD-10] **OR** "depression"[Mesh and ICD-10] **OR** "mania"[Mesh and ICD-10] **OR** "schizophrenia"[Mesh and ICD-10] **OR** "dementia"[Mesh and ICD-10] **OR** "substance abuse”[ICD-10] **OR** “bipolar disorder”[Mesh and ICD-10] **OR** “psychosis”[ICD-10] **OR** “organic disorder”[ICD-10] **OR** “anxiety disorder”[Mesh and ICD-10] **OR** “neurotic disorder”[Mesh and ICD-10] **OR** “neurocognitive disorder”[Mesh and ICD-10] **OR** “Post-traumatic stress disorder”[Mesh and ICD-10]). |


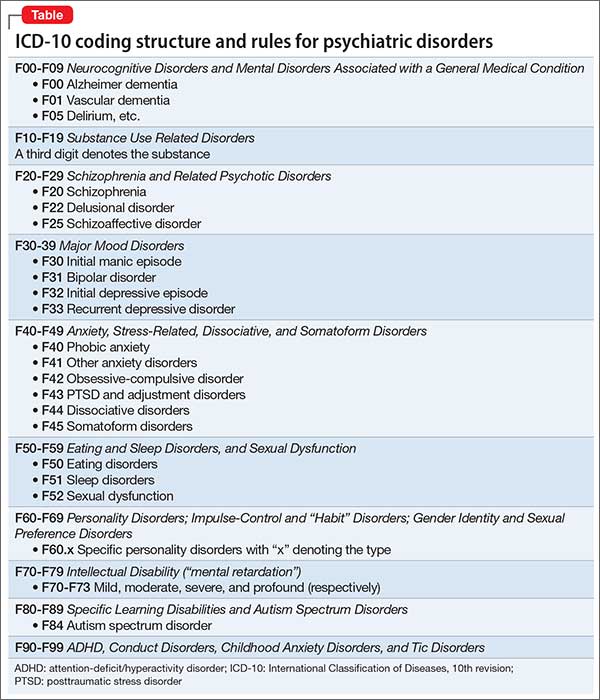


**Figure S4.** International Classification of Diseases 10^th^ revision (ICD-10) with detailed information from the subgroup of the psychiatric chapter. Source: available online <<https://cdn.mdedge.com/files/s3fs-public/images/RTEmagicC_CP015010025_t1.jpg.jpg>>. Access on 10^th^ October, 2022.


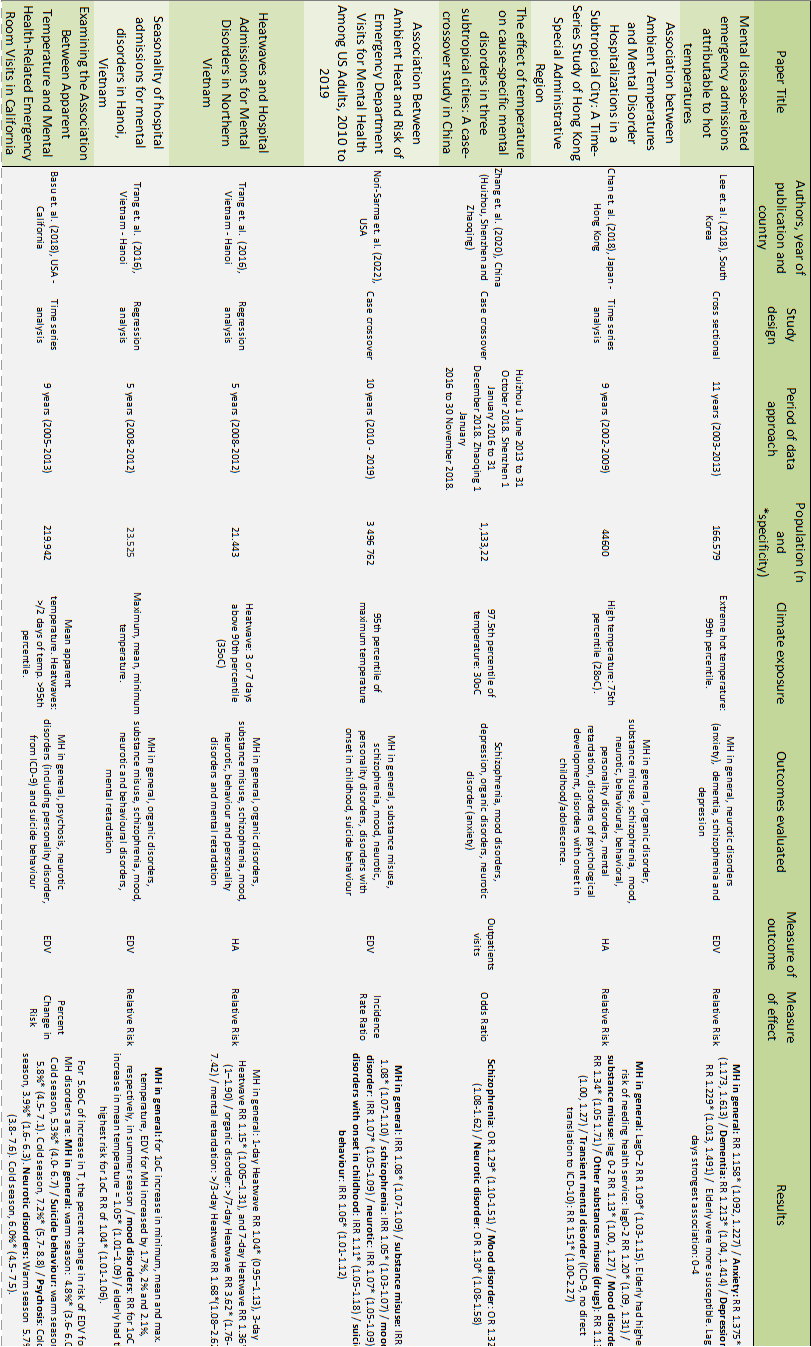


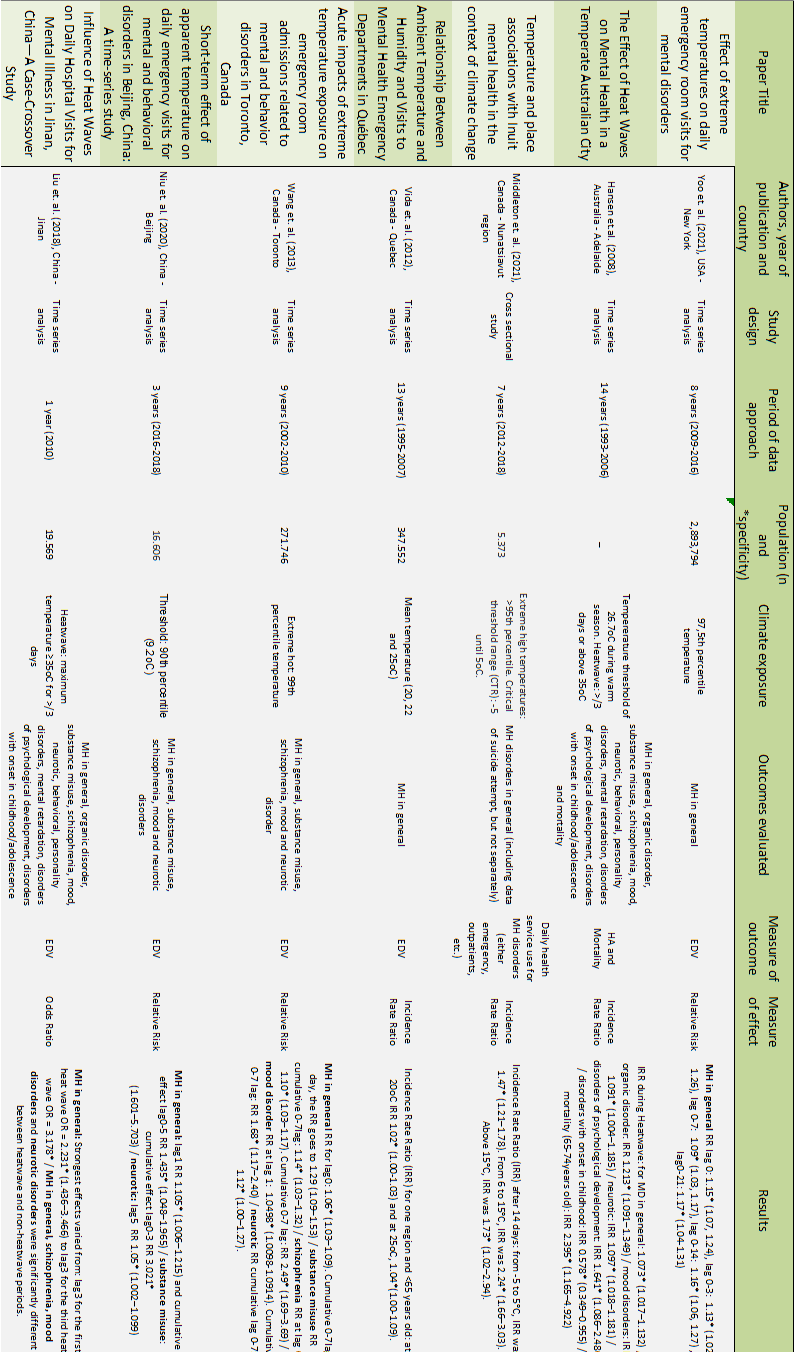


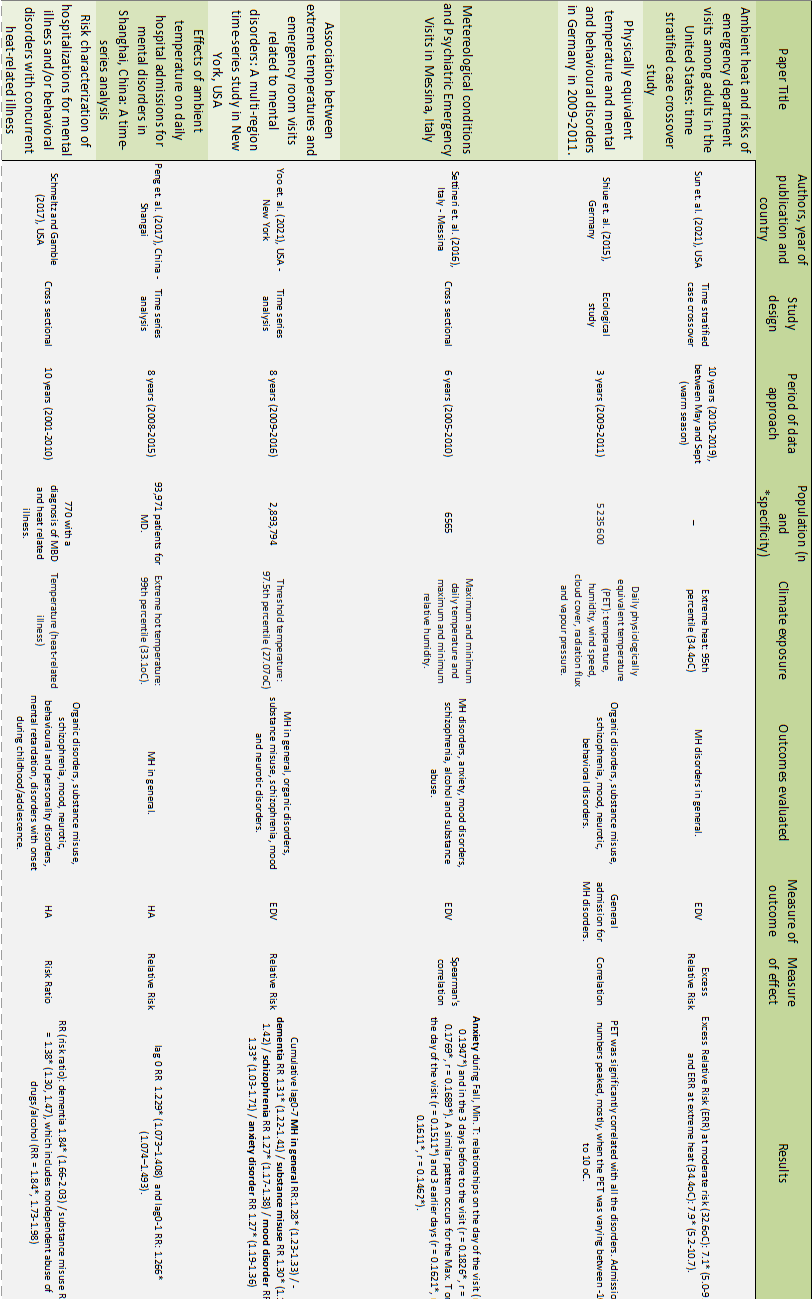


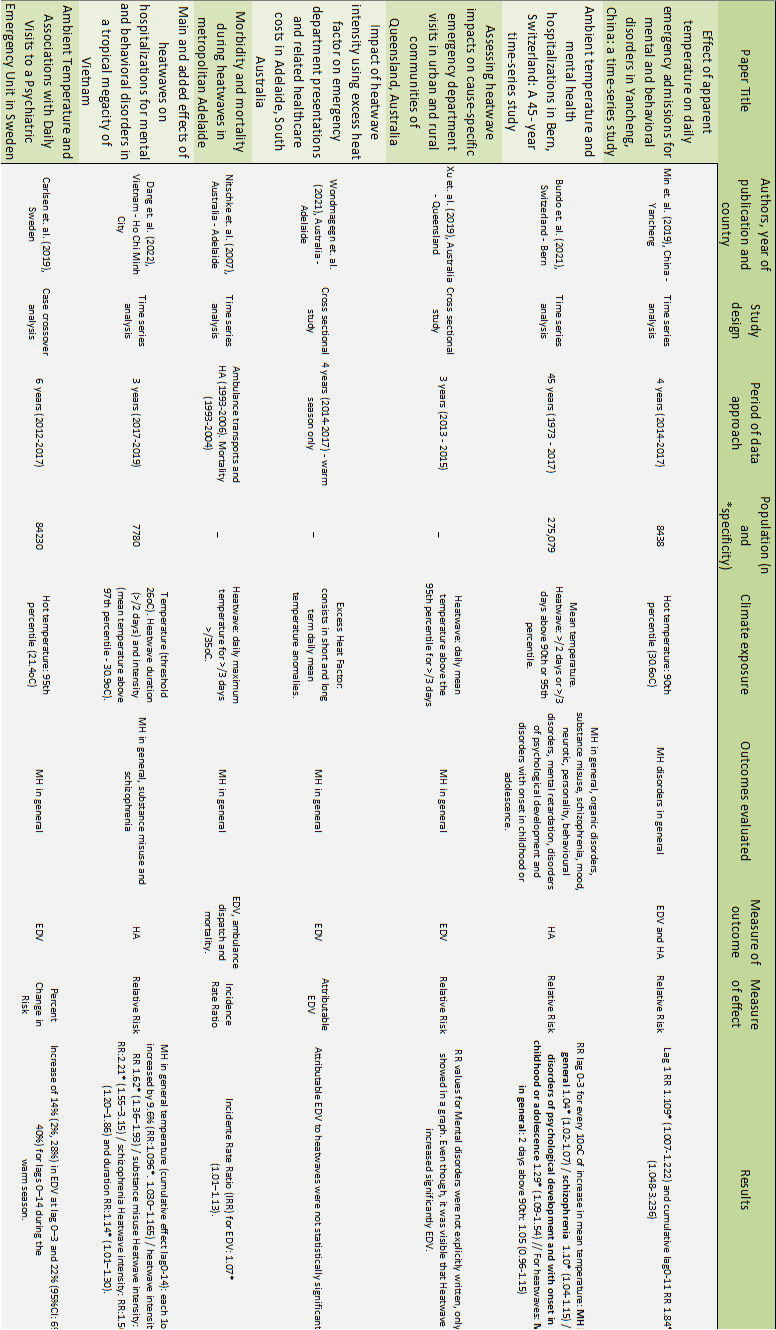


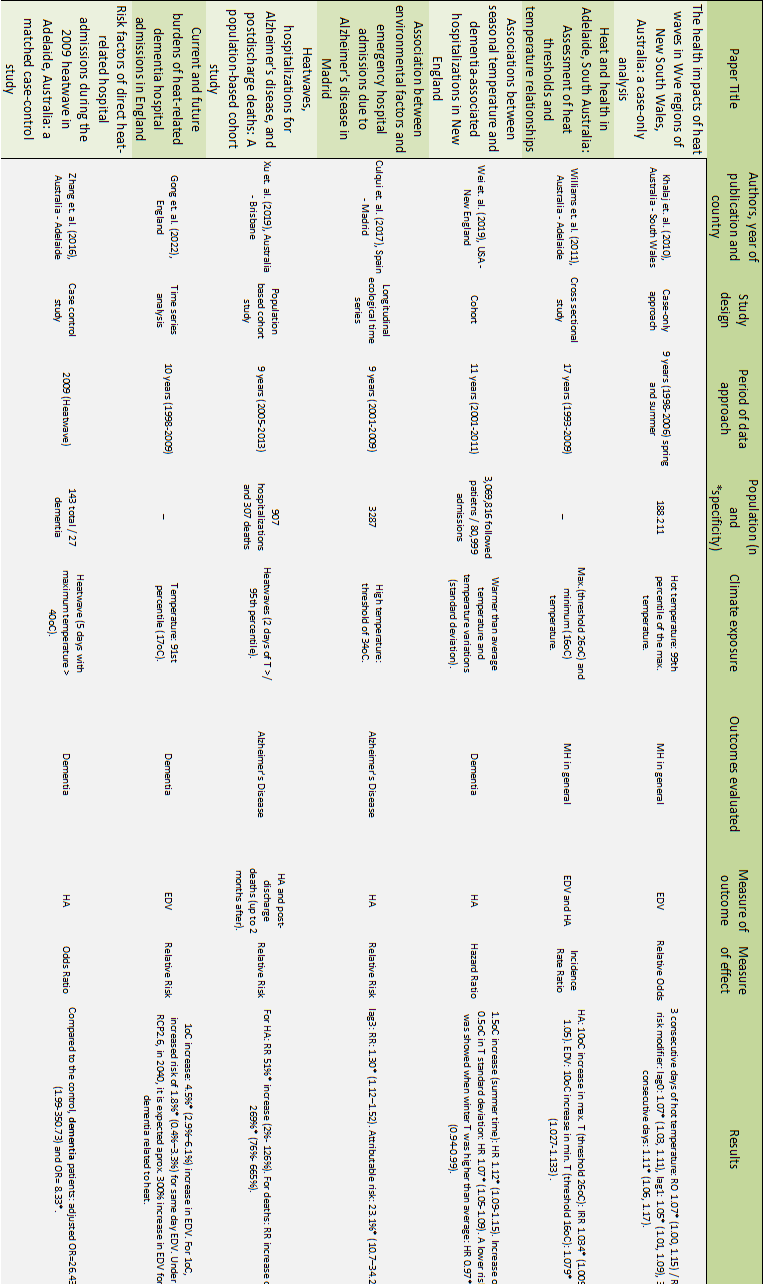


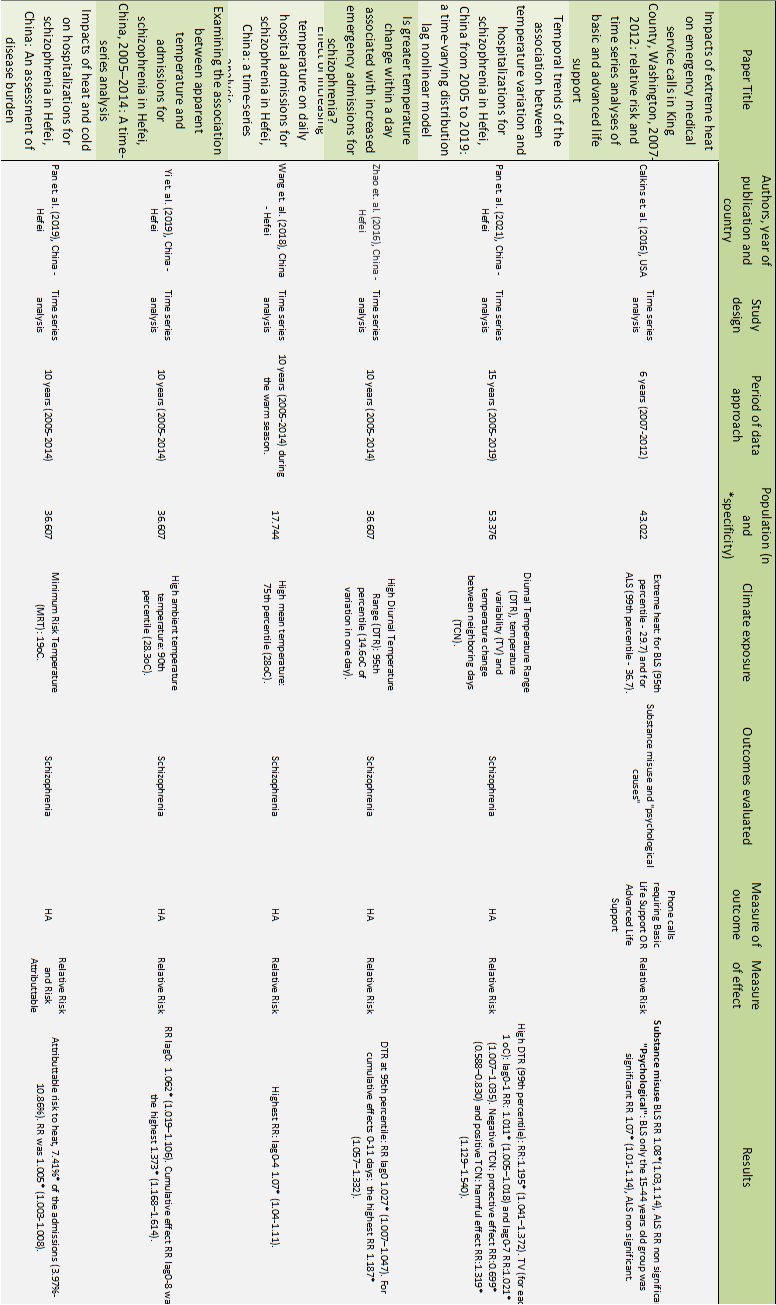


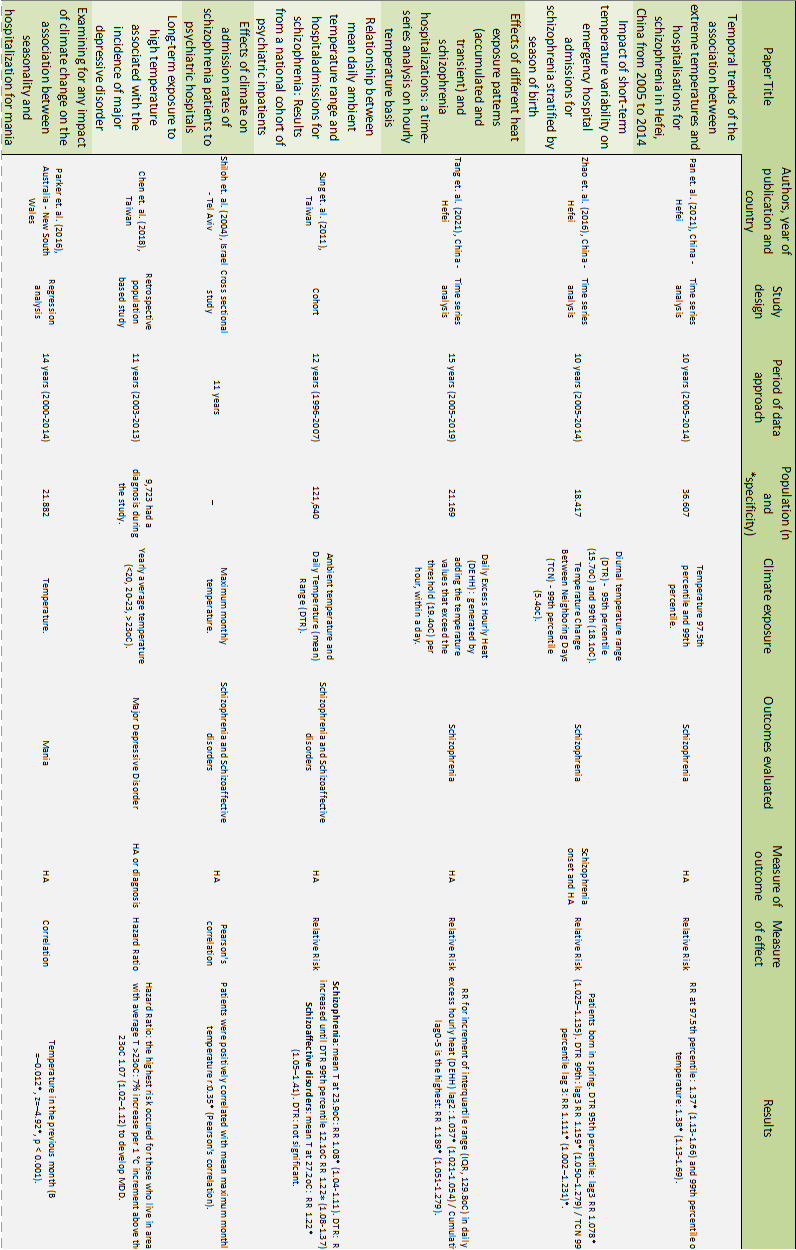


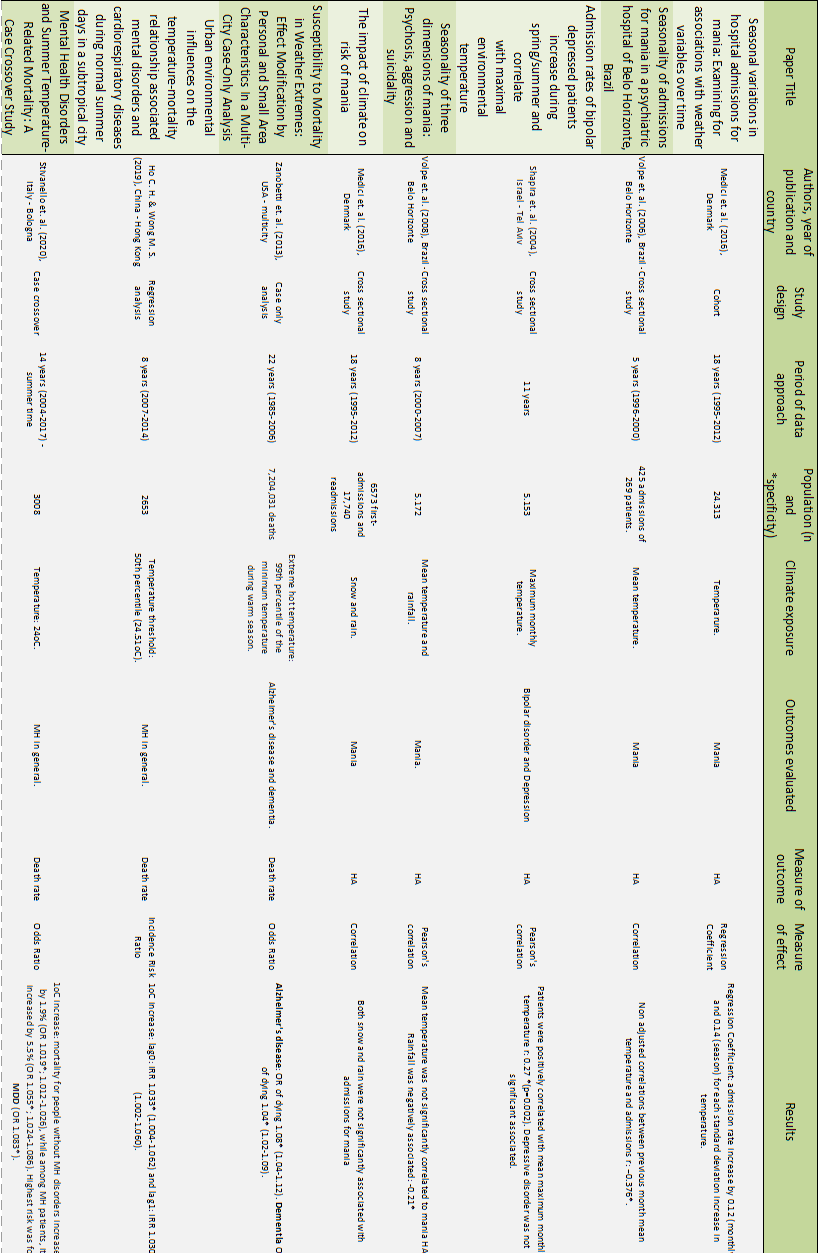


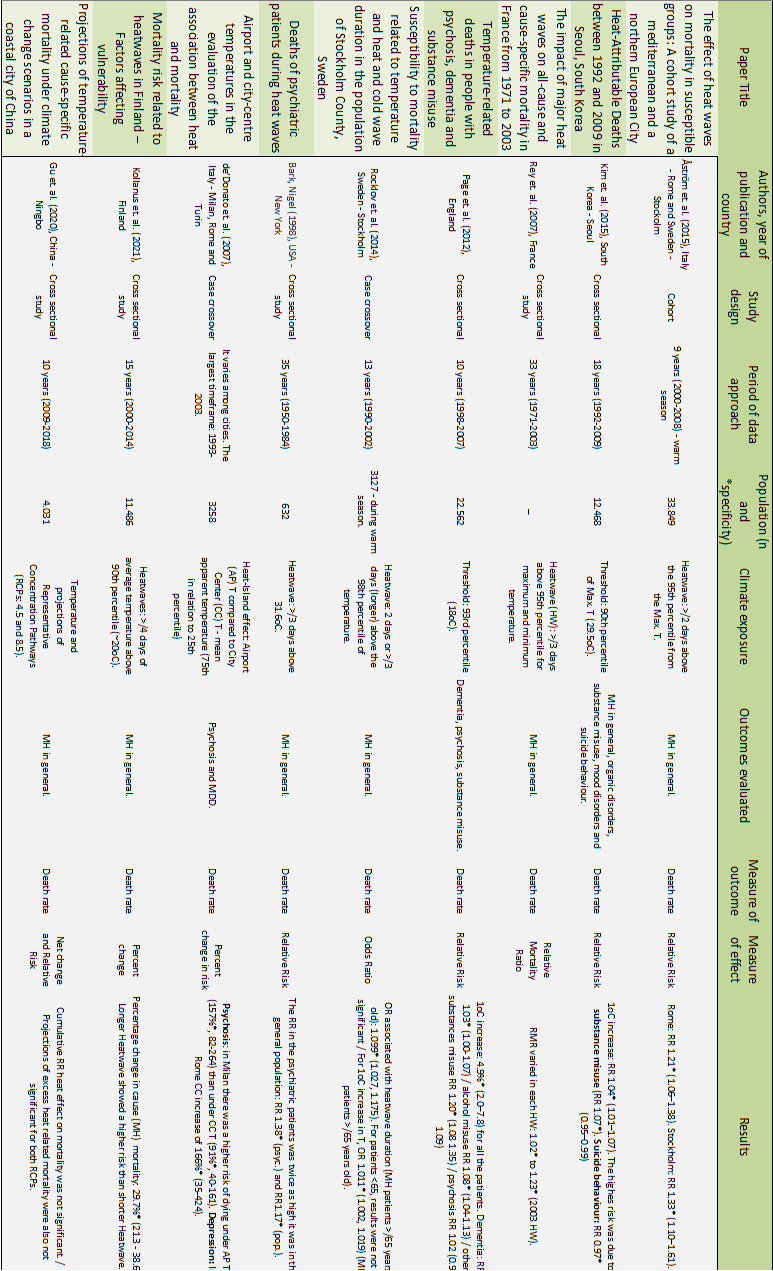


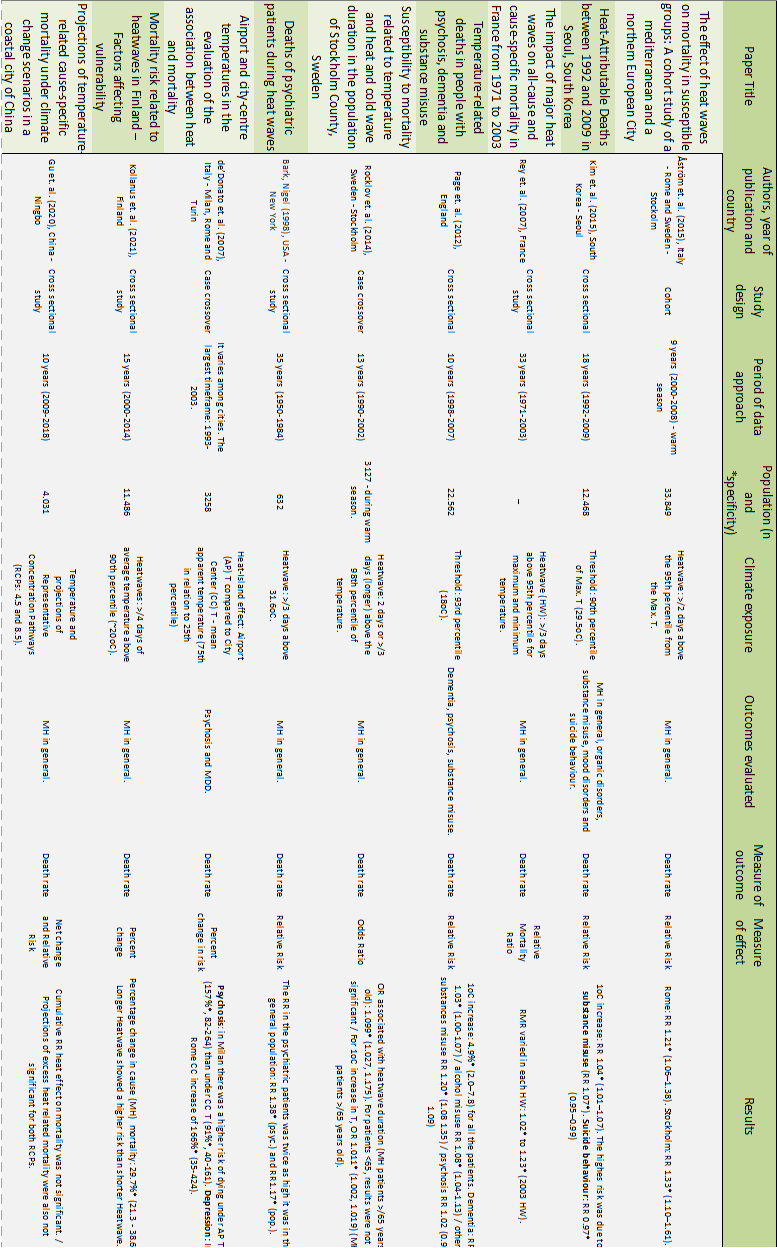


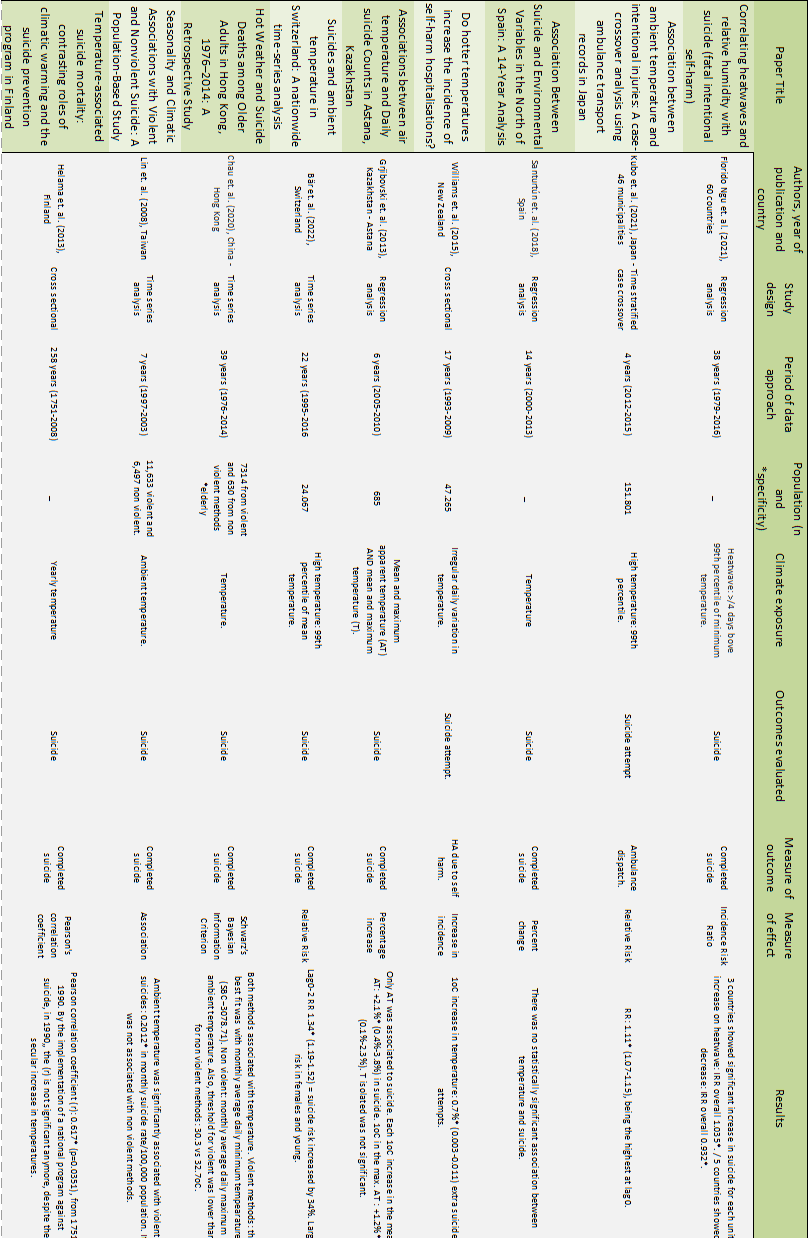


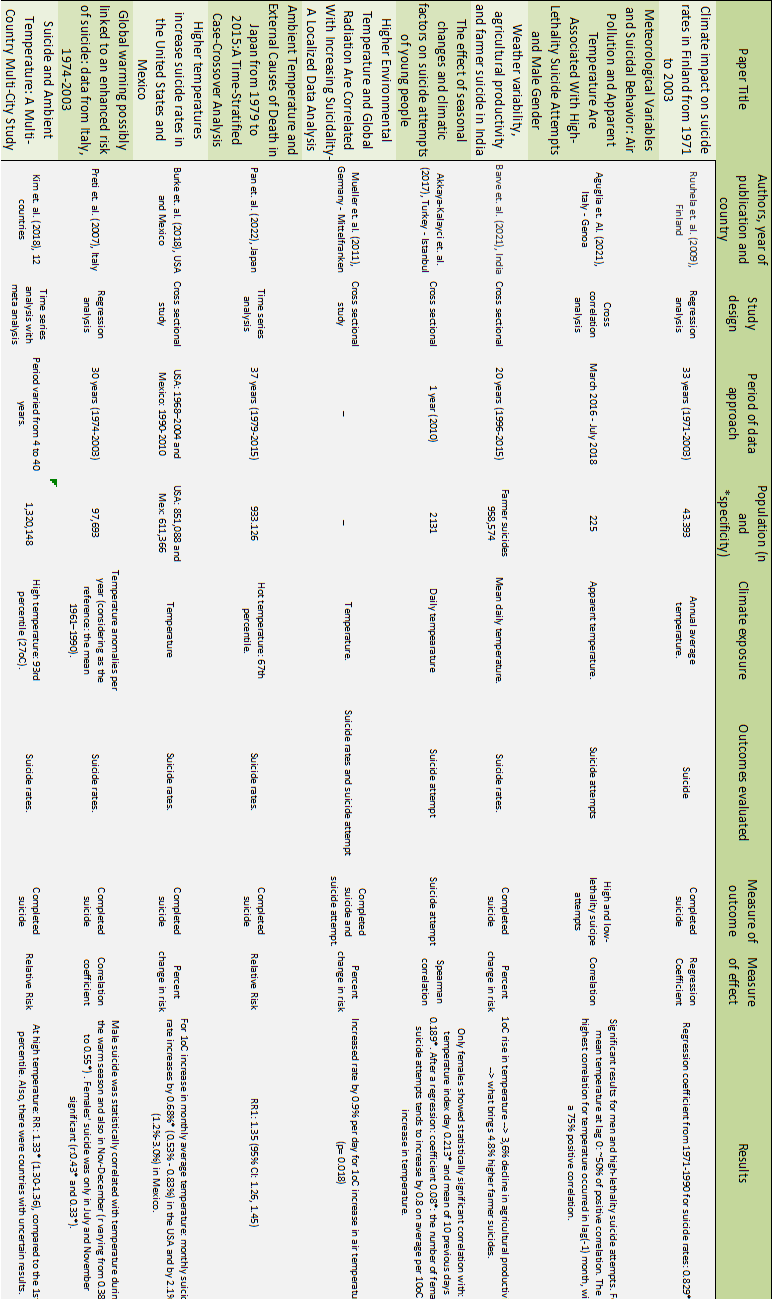


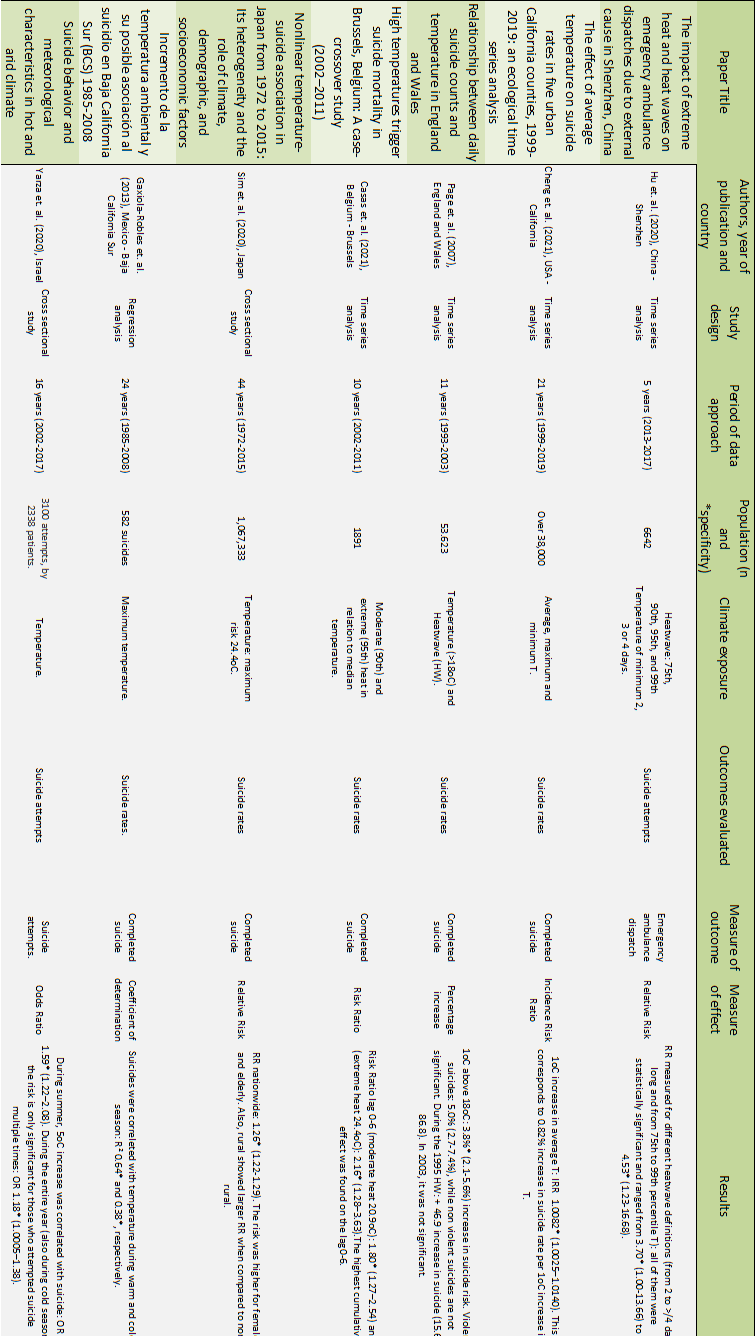


**Figure S5.** With the detailed information from the 105 retrieved studies.

**Table S6.** Quality Assessment Tool for Observational Cohort and Cross-Sectional Studies.

| **Study Quality Assessment Tools** | |
| --- | --- |
| Criteria Yes, No, Other (CD, NR, NA)* | |
| 1. Was the research question or objective in this paper clearly stated?  2. Was the study population clearly specified and defined?  3. Was the participation rate of eligible persons at least 50%?  4. Were all the subjects selected or recruited from the same or similar populations (including the same time period)? Were inclusion and exclusion criteria for being in the study prespecified and applied uniformly to all participants?  5. Was a sample size justification, power description, or variance and effect estimates provided?  6. For the analyses in this paper, were the exposure(s) of interest measured prior to the outcome(s) being measured?  7. Was the timeframe sufficient so that one could reasonably expect to see an association between exposure and outcome if it existed?  8. For exposures that can vary in amount or level, did the study examine different levels of the exposure as related to the outcome (e.g., categories of exposure, or exposure measured as continuous variable)?  9. Were the exposure measures (independent variables) clearly defined, valid, reliable, and implemented consistently across all study participants?  10. Was the exposure(s) assessed more than once over time?  11. Were the outcome measures (dependent variables) clearly defined, valid, reliable, and implemented consistently across all study participants?  12. Were the outcome assessors blinded to the exposure status of participants?  13. Was loss to follow-up after baseline 20% or less?  14. Were key potential confounding variables measured and adjusted statistically for their impact on the relationship between exposure(s) and outcome(s)? | 1. Was the research question or objective in this paper clearly stated and appropriate?  2. Was the study population clearly specified and defined?  3. Did the authors include a sample size justification?  4. Were controls selected or recruited from the same or similar population that gave rise to the cases (including the same timeframe)?  5. Were the definitions, inclusion and exclusion criteria, algorithms or processes used to identify or select cases and controls valid, reliable, and implemented consistently across all study participants?  6. Were the cases clearly defined and differentiated from controls?  7. If less than 100 percent of eligible cases and/or controls were selected for the study, were the cases and/or controls randomly selected from those eligible?  8. Was there use of concurrent controls?  9. Were the investigators able to confirm that the exposure/risk occurred prior to the development of the condition or event that defined a participant as a case?  10. Were the measures of exposure/risk clearly defined, valid, reliable, and implemented consistently (including the same time period) across all study participants?  11. Were the assessors of exposure/risk blinded to the case or control status of participants?  12. Were key potential confounding variables measured and adjusted statistically in the analyses? If matching was used, did the investigators account for matching during study analysis? |
| Quality Rating (Good, Fair, or Poor) | |
| Additional Comments (If POOR, please state why). | |
| *CD, cannot determine; NA, not applicable; NR, not reported | |
| Study Quality Assessment Tool – Quality Assessment for Observational Cohort and Cross-Sectional Studies. Available in <https://www.nhlbi.nih.gov/health-topics/study-quality-assessment-tools> Assessed in 18^th^ March, 2022. | Study Quality Assessment Tool – Quality Assessment for Case-Control Studies. Available in <https://www.nhlbi.nih.gov/health-topics/study-quality-assessment-tools> Assessed in 10^th^ December, 2022. |
